# Survival analysis of immune-related lncRNA in low-grade glioma

**DOI:** 10.1186/s12885-019-6032-3

**Published:** 2019-08-16

**Authors:** Xiaozhi Li, Yutong Meng

**Affiliations:** 1 Department of Neurosurgery, Shengjing Hospital of China Medical University, Shenyang, China; 2 Department of Stomatology, Shengjing Hospital of China Medical University, No. 36 Sanhao Street, Shenyang, Liaoning Province 110004 People’s Republic of China

**Keywords:** Low-grade glioma, lncRNA, Immune, Prognosis

## Abstract

**Background:**

Low-grade glioma is grade I-II glioma. Immunotherapy is a promising way of tumor killing. Research on immune molecular mechanisms in low-grade gliomas and discovery of new immune checkpoints for low-grade gliomas are of great importance.

**Methods:**

Gene sequencing data and clinical data of low-grade glioma were downloaded from TCGA database. Prognosis related lncRNAs were identified by Cox regression and their possible functions were found by gene enrichment set analysis.

**Results:**

A total of 529 low-grade glioma samples and 5 non-tumor brain tissue samples are obtained from the TCGA database. Two hundred forty-seven immune-associated lncRNAs are screened. Cox regression showed that 16 immune-related lncRNAs are associated with low-grade glioma prognosis, and 7 lncRNAs are independent risk factors. Gene set enrichment analysis suggests that these molecules are enriched in extracellular region, sequence-specific DNA binding, neuropeptide signaling pathway, transcriptional misregulation in cancer, cytokine-cytokine receptor interaction, protein digestion and absorption, chemokine signaling pathway, etc.

**Conclusion:**

The identification of immune-related lncRNA may provide new targets for the research of the molecular mechanisms and treatment of low-grade glioma.

**Electronic supplementary material:**

The online version of this article (10.1186/s12885-019-6032-3) contains supplementary material, which is available to authorized users.

## Background

Low-grade glioma is grade I-II glioma, the main components of which are oligodendroglioma and astrocytoma. Prognosis of low-grade glioma is better than high-grade glioma, suggesting that the pathogenesis of low-grade glioma and high-grade glioma is different [1]. The current treatment of low-grade gliomas still tends to be based on surgically based comprehensive treatment [2, 3]. Immunotherapy is a new way of killing tumors. Among them, blockers for the PD-1/PD-L1 pathway have achieved great success in melanoma [4, 5]. Although glioma immunotherapy has a long history, the effect is unsatisfactory [6]. Therefore, the study of immune molecular mechanisms for low-grade gliomas and the discovery of new immune checkpoints are important for the treatment of low-grade gliomas. Long non-coding RNA (lncRNA) is a kind of non-coding RNA of more than 200 nucleotides in length, which is involved in epigenetic regulation, alternative splicing, post-transcriptional regulation and other gene regulation methods in gliomas [7]. This study identified immune-related lncRNAs in low-grade gliomas and explored the relationship between these immune-related lncRNAs and the prognosis of low-grade gliomas.

## Methods

### Acquisition of low-grade glioma expression data

Low-grade glioma non-tumor brain tissue RNA-Seq data (level 3) and clinical data were downloaded from the TCGA (https://cancergenome.nih.gov/) database. “edgeR” package of R software was used to normalize the whole dataset and obtain the differentially expressed genes. |log_2_FC| > 2 and false discovery rate (FDR) < 0.05 were used as threshold. All of these data were retrieved from TCGA database which are open to the public under guidelines, so it is confirmed that all informed consent was achieved.

### Immune-associated lncRNAs

Immune regulatory factor list was downloaded from the InnateDB database (www.innatedb.com). Correlation between the molecules was calculated. lncRNAs with correlation coefficient > 0.7 and *P* < 0.05 were used for further analysis.

### Cox regression

Univariate Cox regression was performed on immune-related lncRNA and clinical survival data to identify prognostic-related lncRNAs (Efron approximation was used). Stepwise regression multivariate Cox analysis was performed to establish a risk score. The risk score is expressed as: risk score = β_gene1_ × Expression_gene1_ + β_gene2_ × Expression_gene2_ + β_gene3_ × Expression_gene3_ + ... + β_genen_ × Expression_genen_. Kaplan-Meier survival curve based on risk scores was drew.

### Gene set enrichment analysis

GO (gene ontology) and KEGG (Kyoto Encyclopedia of Genes and Genomes) enrichment analysis of low-grade glioma immune-related lncRNAs was performed on the DAVID website (https://david.ncifcrf.gov/) to explore potential biological pathways that immune-related lncRNA may be involved in.

### Statistical analysis

R software 3.6.0 was used to conduct all statistical analyses in this study. *P* < 0.05 was considered statistically different. The Pearson correlation test analyzes the correlation between molecules.

## Results

### Differentially expressed lncRNAs in low-grade glioma

**Fig. 1:**
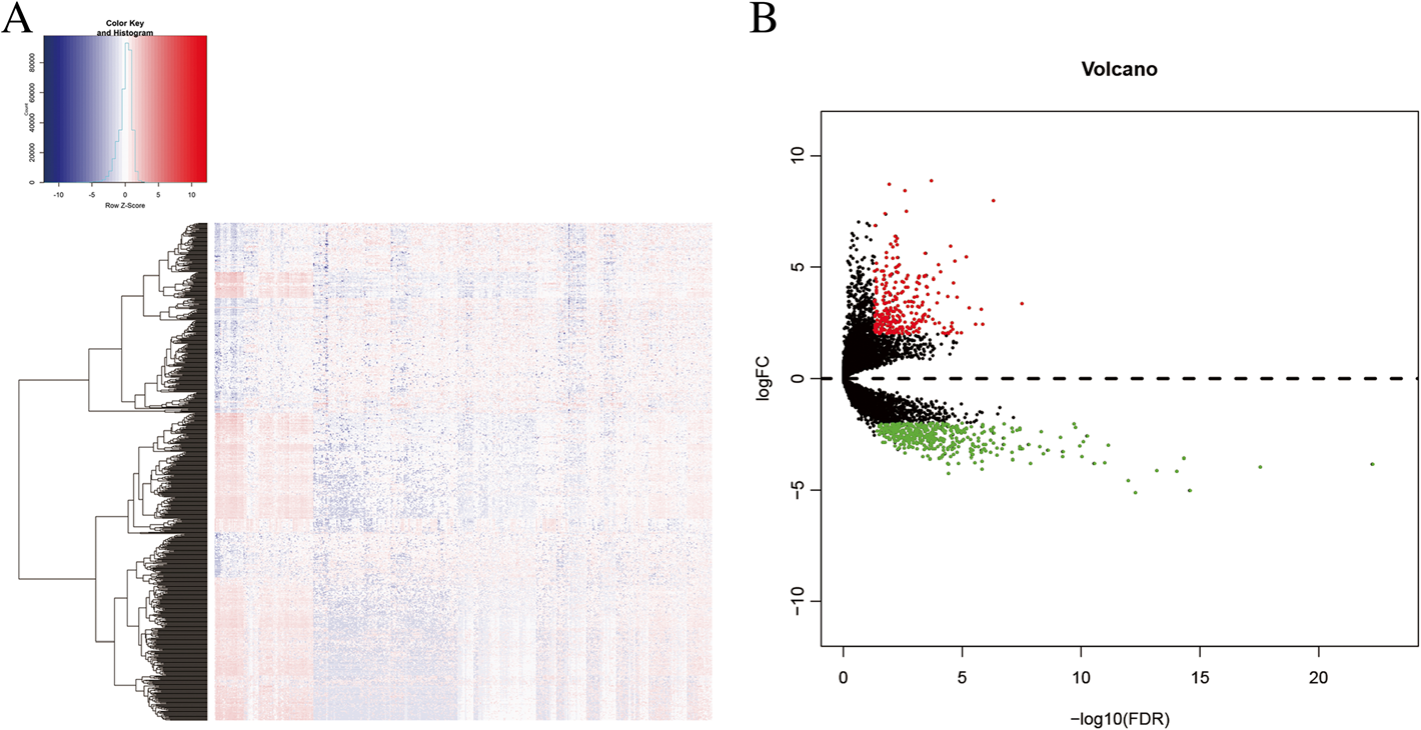
Differential expression lncRNAs. **a** Heatmap of differential expression lncRNAs. **b** Volcano plot of differential expression lncRNAs

A total of 529 low-grade glioma samples and 5 non-tumor brain tissue samples were obtained from the TCGA database. The median age of diagnosis was 41.2 years (14.4–87.1 years). Among them, there are 282 males and 227 females. Three hundred eighty-four patients survived and 125 died at the point of the last follow-up. By contrasting the tumor samples and normal samples, 717 glioblastomas differentially expressed lncRNAs were screened with the threshold of |log_2_FC| > 2 and FDR < 0.05. Among them, 295 lncRNAs expression were up-regulated and 422 lncRNAs expression were down-regulated. The heatmap and volcano map of differentially expressed lncRNAs are shown in Fig. 1.

### Immune-associated lncRNAs in low-grade glioma

**Fig. 2:**
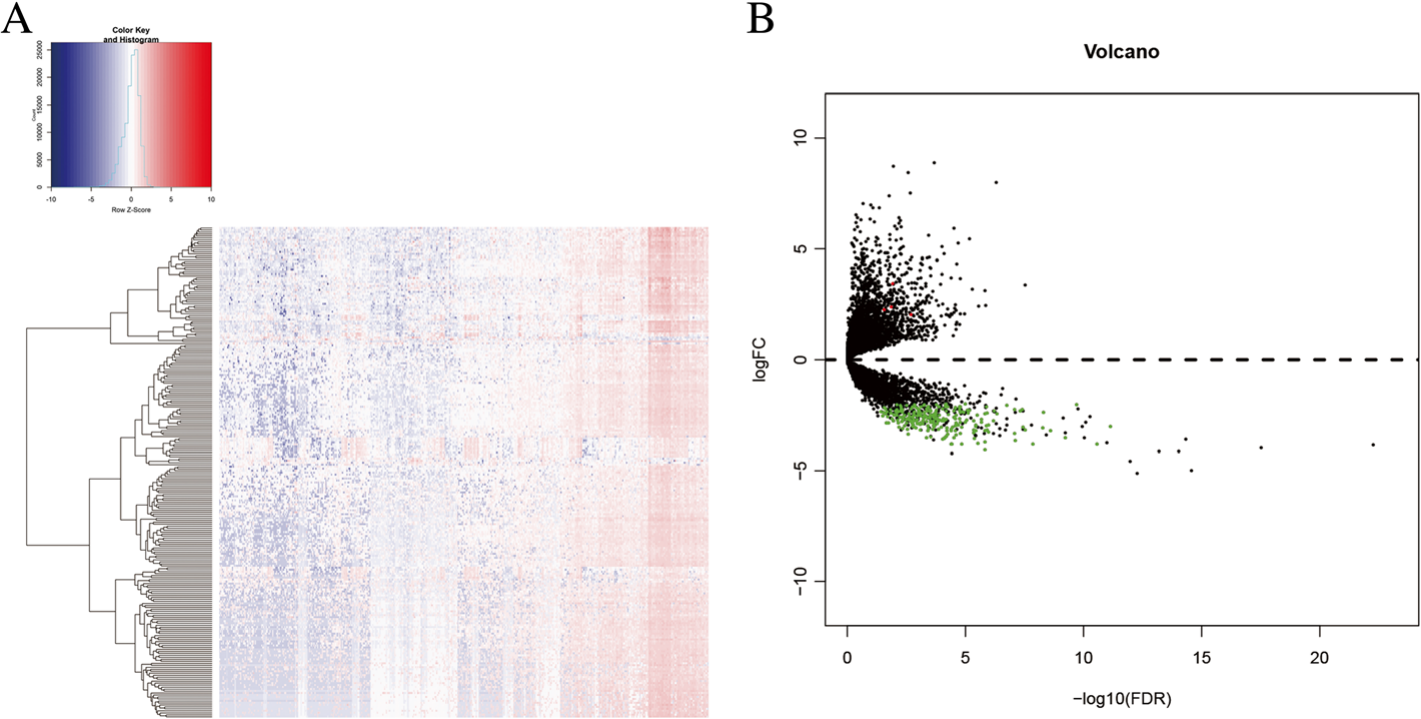
Differential expression immune-associated lncRNAs. **a** Heatmap of differential expression immune-associated lncRNAs. **b** Volcano plot of differential expression immune-associated lncRNAs

The list of immunoregulatory genes was downloaded from the InnateDB database, and we extracted the immunomodulatory genes. Interestingly, we identified more down-regulated immuno-related lncRNAs (242 lncRNAs) than up-regulated immune-related lncRNAs (5 lncRNAs). The heatmap and volcano map of the immune-related lncRNAs in low-grade glioma are shown in Fig. 2.

### Cox regression

**Fig. 3:**
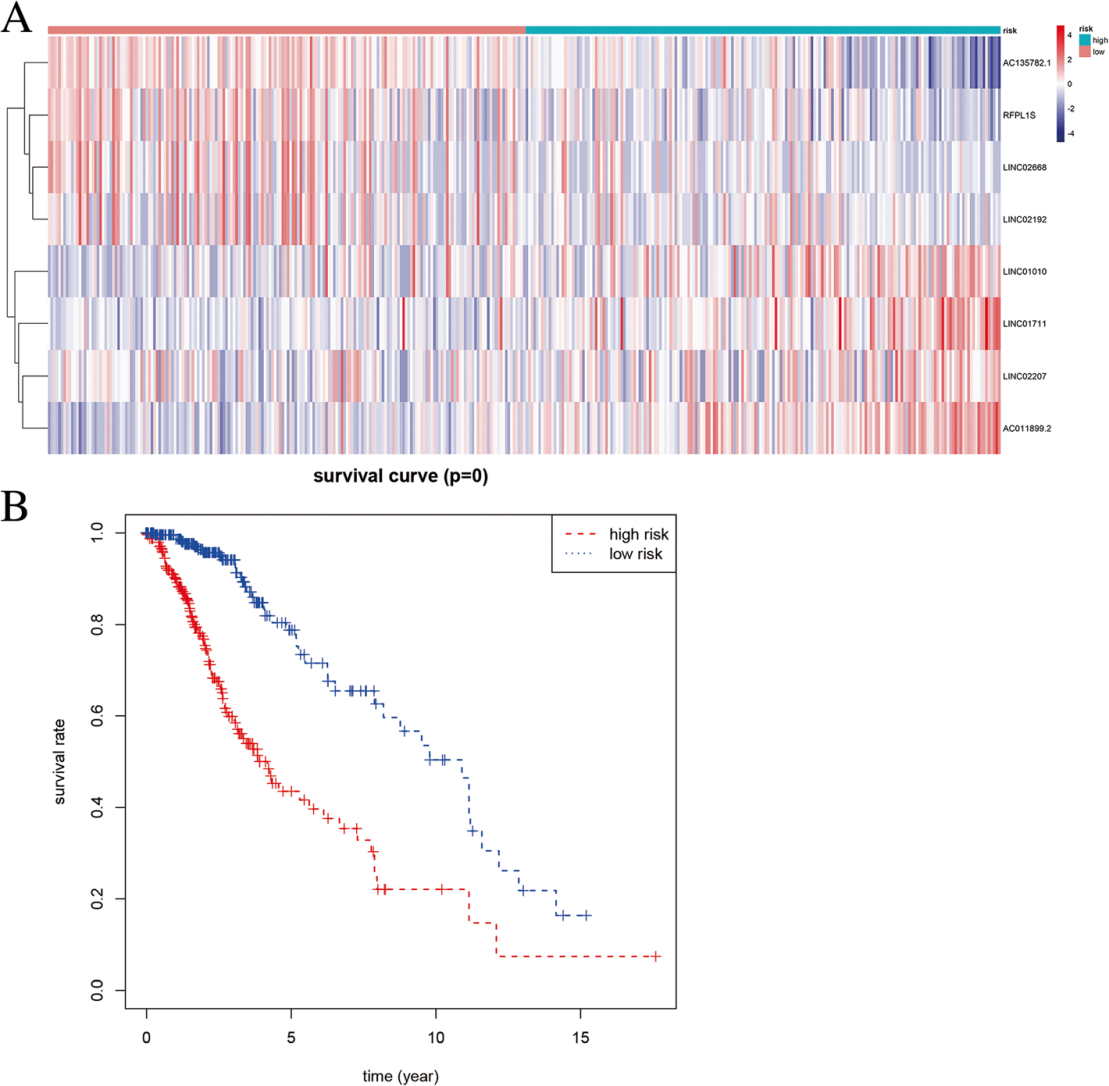
Survival-associated immune mRNAs. **a** Heatmap of survival-associated immune lncRNAs. **b** Kaplan–Meier survival curves for survival-associated immune lncRNAs

We used “caret” package of R language to divide the glioma samples into training cohort and validation cohort by the ratio of 7:3. The expression matrix of 247 immune-related lncRNAs were fused with survival data, and univariate Cox regression was used to analyze the prognostic risk factors of low-grade glioma in the training cohort first. A total of 16 lncRNAs were identified as prognostic risk factors. Stepwise regression multivariate Cox regression was performed to establish the risk score. Eight lncRNAs entered the risk scoring model, risk score = 0.281 * Expression_LINC01010_–0.271 * Expression _AC135782.1_ + 0.214* Expression _LINC01711_–0.196* Expression _RFPL1S_ - 0.262* Expression _LINC02668_–0.143* Expression _LINC02207_ + 0.289* Expression _AC011899.2_ + 0.319 * Expression _LINC02192_. Among the 8 lncRNAs, 7 immune-related lncRNAs were independent prognostic risk factors for low-grade glioma. The results of the univariate and multivariate Cox regression models are shown in Table 1. The 8-lncRNAs heatmap involved in constructing the risk scoring model are shown in Fig. 3a. According to the median value of the risk score, low-grade glioma patients were divided into high-risk group and low-risk group in the training cohort. We found that the overall survival time of patients in the high-risk group was much lower than that in the low-risk group (as shown in Fig. 3b).

**Table 1:** Univariate analysis and multivariate analysis of immune-associated lncRNAs

lncRNA	Univariate Analysis		Multivariate Analysis	
	HR (95%CI)	*P*	HR (95%CI)	*P*
LINC01010	1.018	0.003	1.325	0.001
AC135782.1	0.995	0.030	0.763	0.002
LINC01711	1.001	0.041	1.238	0.001
RFPL1S	1.000	0.029	0.822	0.029
LINC02668	0.980	0.023	0.770	0.010
LINC02207	1.014	0.024	0.867	0.109
AC011899.2	1.004	< 0.001	1.335	0.003
LINC02192	0.991	0.037	1.375	< 0.001

The predicting performance of the 8-lncRNAs model was calculate in both training cohort and validation cohort by the area under ROC (Receiver operating characteristic) curve (AUC). The ROC curve had a 3-year survival AUC area of 0.845 and a 5-year survival AUC area of 0.746 in the training cohort while The ROC curve had a 3-year survival AUC area of 0.810 and a 5-year survival AUC area of 0.738 in the training cohort, as shown in Additional file 1: Figure S1.

### Gene set enrichment analysis

**Fig. 4:**
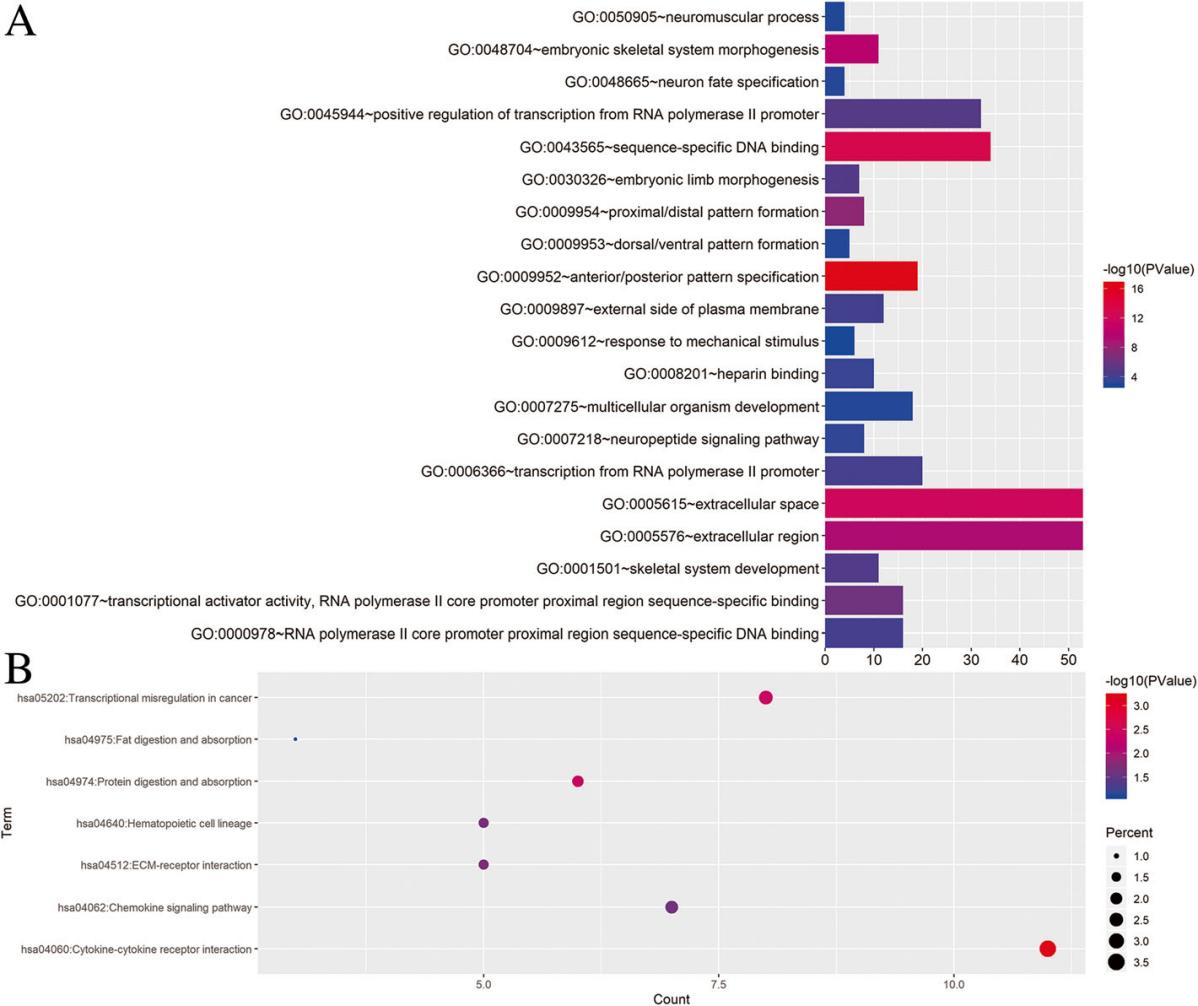
Functional enrichment analysis. **a** GO biological process enrichment results. **b** KEGG biological process enrichment results

GO and KEGG enrichment analysis were performed on the differentially expressed gene sets of the above high-risk and low-risk groups. The results are shown in Fig. 4. Taking *P* < 0.05 as the statistical threshold, GO enrichment analysis indicated that the genes were enriched in extracellular region, sequence-specific DNA binding, neuropeptide signaling pathway, etc. KEGG enrichment analysis suggested that these genes were involved in transcriptional misregulation in cancer, cytokine-cytokine receptor interaction, protein digestion and absorption, chemokine signaling pathway, etc. These enriched items may help scientists and doctors determine the directions of further research of the mechanisms by which immune-related lncRNAs affecting glioma.

## Discussion

Targeted therapy for immune checkpoints is one method of tumor immunotherapy. Immune regulation against immune checkpoints can lead to tumor cell death by providing immune response signals to T cells [8]. Classical tumor immune checkpoints include PD-1, PD-L1, PD-L2 and CTLA-4. So far, ipilimumab (CTLA-4 blocking antibody), and Pembrolizumab and Nivolumab (PD-1 blocking antibody) have been approved by the FDA. Satisfactory results are presented in the treatment of melanoma [9]. However, there are no effective immune checkpoints for the treatment of glioma [10]. For instance, immunohistochemistry experiments show that PD-L1 appears to be highly expressed only in grade IV gliomas [11]. There is a strong need to screen and research of new immune checkpoints for low-grade glioma.

SCHLAP1 is one of the low-grade glioma immune-related lncRNAs we screened. It has been reported that SCHLAP1 is up-regulated in prostate cancer compared with benign prostatic hyperplasia and normal tissue [12–16]. SCHLAP1 promotes proliferation and metastasis of prostate cancer by targeting miR-198 and promoting MAPK1 pathway [17]. In bladder cancer, SCHLAP1 acts as a pro-oncogene, and silencing SCHLAP1 induces proliferation of bladder cancer cells, promotes apoptosis, and inhibits cell migration [18]. In addition, CALML3-AS1 is also one of the low-grade glioma immune-related lncRNAs we screened, and it has been reported to inhibit microRNA-4316 in bladder cancer, thereby upregulating ZBTB2 and promoting tumorigenesis of bladder cancer [19].

## Conclusion

This study identified 247 immune-related lncRNAs in low-grade glioma. Cox regression analysis showed that 16 lncRNAs were associated with prognosis in patients with low-grade glioma, and 7 lncRNAs were independent prognostic risk factors. Gene set enrichment analysis revealed that these immune-related lncRNAs may be involved in functions such as extracellular region, sequence-specific DNA binding, neuropeptide signaling pathway, transcriptional misregulation in cancer, cytokine-cytokine receptor interaction, protein digestion and absorption, chemokine signaling pathway, etc. The identification of immune-related lncRNA may provide new targets for the research of the molecular mechanisms and treatment of low-grade glioma.

## Additional file

Additional file 1: **Figure S1.** Evaluation of prognostic performance of the model. (A) ROC curves of training cohort. (B) ROC curves of validation cohort. (TIF 696 kb)

## Data Availability

This study obtained open data from the TCGA database. (https://cancergenome.nih.gov/).

## Abbreviations

AUC: The area under ROC curve
GO: Gene ontology
KEGG: Kyoto Encyclopedia of Genes and Genomes
lncRNA: Long non-coding RNA
ROC: Receiver operating characteristic
