# Supplementary figures and images for: Survival analysis of immune-related lncRNA in low-grade glioma
Source: BMC Cancer. 2019 Aug 16;19:813. doi: 10.1186/s12885-019-6032-3 (PMC6697914; doi:10.1186/s12885-019-6032-3)

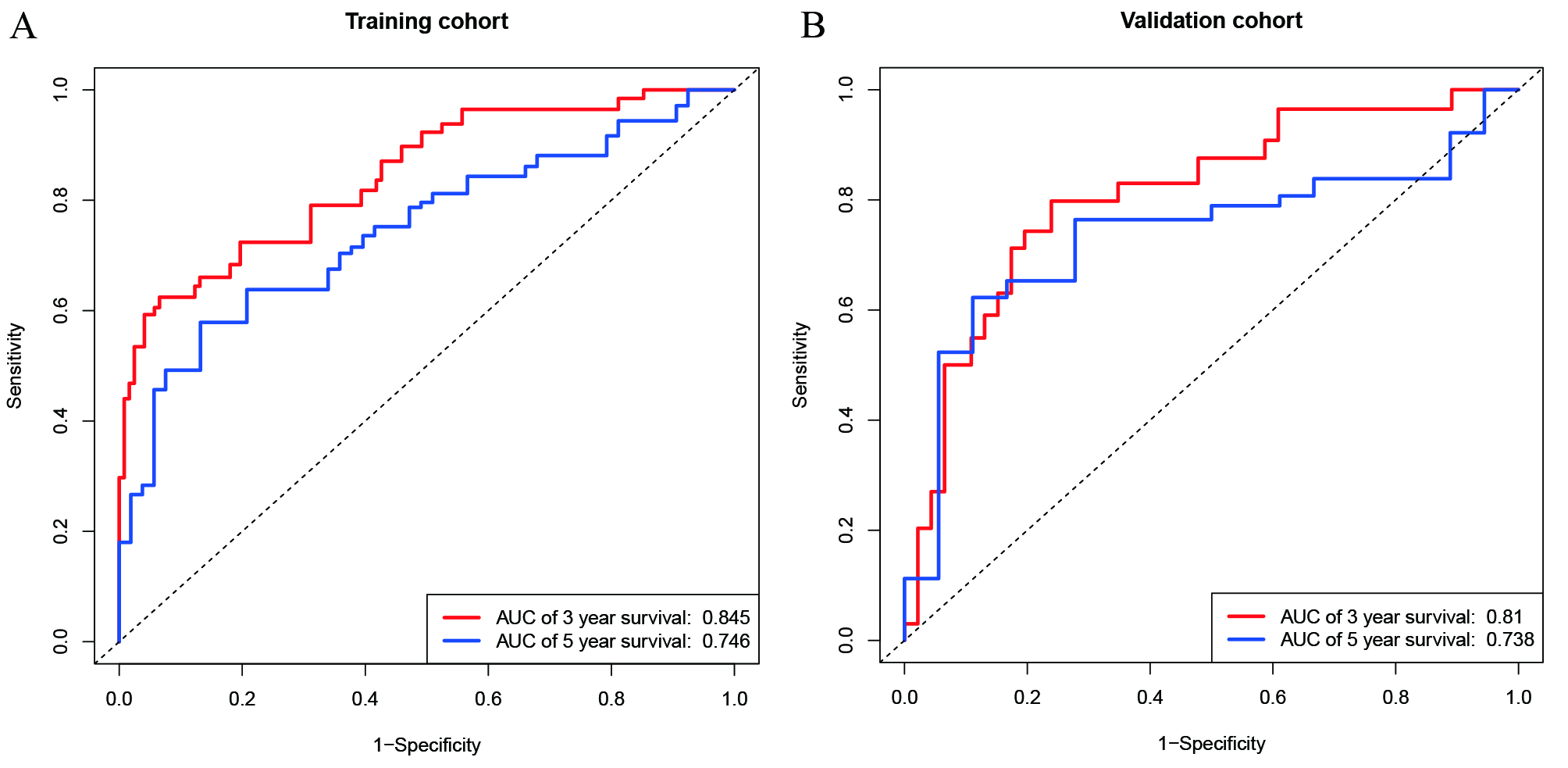

Supplement: Supplementary file 1 — Figure S1. Evaluation of prognostic performance of the model. (A) ROC curves of training cohort. (B) ROC curves of validation cohort. (TIF 696 kb) [file 12885_2019_6032_MOESM1_ESM.tif]
